# Sarcomere Disassembly and Transfection Efficiency in Proliferating Human iPSC-Derived Cardiomyocytes

**DOI:** 10.3390/jcdd9020043

**Published:** 2022-01-27

**Authors:** Qianliang Yuan, Renee G. C. Maas, Ellen C. J. Brouwer, Jiayi Pei, Christian Snijders Blok, Marko A. Popovic, Nanne J. Paauw, Niels Bovenschen, Jesper Hjortnaes, Magdalena Harakalova, Pieter A. Doevendans, Joost P. G. Sluijter, Jolanda van der Velden, Jan W. Buikema

**Affiliations:** 1 Amsterdam Cardiovascular Sciences, Department of Physiology, Amsterdam University Medical Centers, De Boelelaan 1108, 1081 HZ Amsterdam, The Netherlands; q.yuan@amsterdamumc.nl (Q.Y.); e.c.j.brouwer@student.vu.nl (E.C.J.B.); j.vandervelden1@amsterdamumc.nl (J.v.d.V.); 2 Utrecht Regenerative Medicine Center, Circulatory Health Laboratory, University Utrecht, 3584 CS Utrecht, The Netherlands; r.g.c.maas-4@umcutrecht.nl (R.G.C.M.); j.pei-2@umcutrecht.nl (J.P.); c.j.b.Snijdersblok-5@umcutrecht.nl (C.S.B.); m.harakalova@umcutrecht.nl (M.H.); p.doevendans@umcutrecht.nl (P.A.D.); j.sluijter@umcutrecht.nl (J.P.G.S.); 3 Department of Cardiology, University Medical Center Utrecht, Heidelberglaan 100, 3584 CX Utrecht, The Netherlands; 4 Department of Molecular Cell Biology and Immunology (MCBI), Amsterdam University Medical Centers, De Boelelaan 1108, 1081 HZ Amsterdam, The Netherlands; m.popovic@amsterdamumc.nl (M.A.P.); nj.paauw@amsterdamumc.nl (N.J.P.); 5 Bachelor Research Hub, Educational Center, University Medical Centre Utrecht, 3584 CX Utrecht, The Netherlands; n.bovenschen@umcutrecht.nl; 6 Department of Pathology, University Medical Center Utrecht, 3584 CX Utrecht, The Netherlands; 7 Center for Translational Immunology, University Medical Center Utrecht, 3584 CX Utrecht, The Netherlands; 8 Department of Cardiothoracic Surgery, Heart & Lung Center, Leiden University Medical Center, Leiden University, Albinusdreef 2, 2333 ZA Leiden, The Netherlands; j.hjortnaes@lumc.nl; 9 Netherlands Heart Institute, Holland Heart House, Moreelsepark 1, 3511 EP Utrecht, The Netherlands

**Keywords:** iPSC-derived cardiomyocytes, human iPSC, sarcomere development, sarcomere disassembly, transfection efficiency, non-viral vector incorporation, cardiomyocytes, mitosis, M-phase, proliferation, cardiomyocyte proliferation, binucleation, self-duplication

## Abstract

Contractility of the adult heart relates to the architectural degree of sarcomeres in individual cardiomyocytes (CMs) and appears to be inversely correlated with the ability to regenerate. In this study we utilized multiple imaging techniques to follow the sequence of sarcomere disassembly during mitosis resulting in cellular or nuclear division in a source of proliferating human pluripotent stem cell-derived cardiomyocytes (hiPSC-CMs). We observed that both mono- and binuclear hiPSC-CMs give rise to mononuclear daughter cells or binuclear progeny. Within this source of highly proliferative hiPSC-CMs, treated with the CHIR99021 small molecule, we found that Wnt and Hippo signaling was more present when compared to metabolic matured non-proliferative hiPSC-CMs and adult human heart tissue. Furthermore, we found that CHIR99021 increased the efficiency of non-viral vector incorporation in high-proliferative hiPSC-CMs, in which fluorescent transgene expression became present after the chromosomal segregation (M phase). This study provides a tool for gene manipulation studies in hiPSC-CMs and engineered cardiac tissue. Moreover, our data illustrate that there is a complex biology behind the cellular and nuclear division of mono- and binuclear CMs, with a shared-phenomenon of sarcomere disassembly during mitosis.

## 1. Introduction

The contractile tissue of the heart is composed of a mixture of different cells, including approximately 35% cardiomyocytes (CMs) [1]. Proper contraction of CMs is dictated by the architecture of the sarcomeres and appears together with polyploidy inversely correlated with self-renewal. Higher sarcomere architectural organization is related to maturity of the heart [2], while immature organization of sarcomeres correlates to the capacity of CMs to proliferate [3]. Previous studies in rodent hearts have shown that during embryonic and early postnatal hyperplastic growth phases the CMs gradually disassemble their sarcomeres during mitosis to ensure cytokinesis [4,5,6].

Unlike in fish [7], mammals display hyperplastic growth shortly after birth, which is subsequentially silenced leading to heart growth predominantly originating from cellular hypertrophy [8]. The hypertrophic growth dynamic is accompanied by increased ploidy and multinucleation of the adult heart [9,10]. The exact dynamics of human CM multinucleation and self-duplication in relation to sarcomere architecture remain largely unknown. Moreover, reliable determination of CM cytokinesis, multinucleation, self-duplication and nuclear ploidy is challenging [11,12,13].

The advances in human-induced pluripotent stem cells (hiPSCs) biology allow for efficient directed differentiation into CMs (hiPSC-CMs), thereby relying on biphasic Wnt signal modulation and resulting in up to 95% pure populations of hiPSC-CMs [14,15,16]. Currently, hiPSC-CMs are widely used in mechanistic studies, drug testing and tissue engineering [17,18]. We and others demonstrated that a third phase of Wnt signal modulation in immature CMs results in a maturation block while prolongating the window for CM proliferation [3,19,20,21,22]. Using this strategy, it now becomes feasible to maintain 30–50% of Ki67 positive cells and up to 10% of mitotic CMs after day 12 of hiPSC-CM differentiation [3,21]. Here we utilized live imaging in a hiPSC-CM culture system to follow the sequence of sarcomere breakdown during the mitotic phases of CM cell division. We observed that cytokinesis in hiPSC-CMs originates from mononuclear and binuclear CMs and for both routes of duplication the sarcomere breakdown was present during the mitotic phases. Moreover, we observed that a mitotic cell figure, suggestive for the sequence of sarcomere breakdown, was also present during the process of binucleation.

Molecular genetic modification in hiPSC-CMs represents an essential tool for the mechanistic validations and functions of genes and proteins, but is limited by technical challenges to transfect low-proliferative hiPSC-CMs [23]. Here, we show that Wnt activation in hiPSC-CMs results in sarcomere breakdown during mitosis and that increased cell cycle activity facilitates increased efficiency of non-viral vector incorporation. These findings give an insight in the regulation of sarcomere homeostasis during mitotic cell phases and provide a tool for further molecular and engineering studies.

## 2. Methods

### 2.1. Maintenance of hiPSC

hiPSC obtained from the Stanford Cardiovascular Institute Biobank (SCVI-111 and SCVI-273) were non-enzymatically passaged using 0.5 mM EDTA (Thermo Fisher, Waltham, MA, USA) every 4 days as previously described [3]. In brief, cells were incubated with 0.5 mM EDTA-PBS at room temperature for 5–8 min until cells began to separate uniformly throughout the colonies. PBS-EDTA was removed and hiPSC colonies were washed off swiftly using 1 mL E8 complete medium (Thermo Fisher, Waltham, MA, USA). hiPSC clumps were passaged in a ratio of 1:10 routinely at 80% confluence. To improve cell survival, split ratio reliability and to reduce selective pressure, 10 µM of ROCK inhibitor (Y-27632, Stemcell Technologies, Vancouver, BC, Canada) was added to the E8 complete medium for the first 24 h after cell passaging. Matrigel (Corning, Somerville, MA, USA, 1:400) coated plates (Corning, Somerville, MA, USA) were used for hiPSC culture and PSC Cryopreservtion Kit (Gibco, Waltham, MA, USA) was used for the cryopreservation of hiPSC.

### 2.2. Differentiation of hiPSC into Cardiomyocytes

To obtain hiPSC-derived CMs, hiPSCs were grown to ~90% confluence in a 6-well format. hiPSC cells were maintained in E8 complete medium for at least three passages before starting cardiac lineage differentiations. The cells were efficiently differentiated as previously described, using the RPMI/B27 minus insulin medium (Gibco, Waltham, MA, USA) supplemented with 7–8 µM CHIR99021 (cell line specific) (SelleckChem, Houston, TX, USA) for the first 2 days and 2 µM Wnt-C59 (SelleckChem, Houston, TX, USA) for another 48 h. The proliferation of hiPSC-CMs can be conducted by the removal of cell–cell contacts and small-molecule inhibition with CHIR99021 [3]. Subsequential multiple low-density passaging and reintroduction of 2–3 µM CHIR99021 (cell line specific) to the RPMI/B27 media results in an expansion of hiPSC-CMs [3].

### 2.3. Metabolic Maturation of iPSC-CMs

In order to mature the CMs, we used a previously described metabolic maturation medium [24]. After re-plating and purification, the RPMI/B27 medium was replaced by the maturation medium for a minimum of 3 weeks. Metabolic maturation medium was composed in RPMI 1640 (Thermo Fisher Scientific, Waltham, MA, USA, 11875119) with B27 supplement (Thermo Fisher Scientific, Waltham, MA, USA, 17504-044). Maturation media was composed in DMEM without glucose (Thermo Fisher Scientific, Waltham, MA, USA, 11966025) supplemented with 3 mM glucose (Sigma Aldrich, Saint Louis, MO, USA, G7021), 10 mM L-lactate (Sigma Aldrich, Saint Louis, MO, USA, 71718), 5 μg/mL Vitamin B12 (Sigma Aldrich, Saint Luis, MO, USA, V6629), 0.82 μM Biotin (Sigma Aldrich, Saint Louis, MO, USA, B4639), 5 mM Creatine monohydrate (Sigma Aldrich, Saint Louis, MO, USA, C3630), 2 mM Taurine (Sigma Aldrich, Saint Louis, MO, USA, T0625), 2 mM L-carnitine (Sigma Aldrich, Saint Louis, MO, USA, C0283), 0.5 mM Ascorbic acid (Sigma Aldrich, Saint Louis, MO, USA, A8960), 1 × NEAA (Thermo Fisher Scientific, Waltham, MA, USA, 11140), 0.5% (*w*/*v*) Albumax (Thermo Fisher Scientific, Waltham, MA, USA, 11020021), 1 × B27 and 1% KOSR (Thermo Fisher Scientific, Waltham, MA, USA, 10828028). The medium was refreshed every 3–4 days.

### 2.4. Immunofluorescence

hiPSC-CMs after two days of expansion were seeded into a µ-Plate 96 well (Ibidi) with a density of ~5 × 10^4^ cells/well for continuously culturing with RPMI/B27 and RPMI/B27 supplemented with CHIR99021 (3 µM) for six days. Media was refreshed every other day. At day 6, the cells were washed and fixed with 4% formaldehyde for 10 min at room temperature, permeabilized with 0.25% PBS-Triton for 5 min and then blocked with 10% donkey serum for 30 min. The cells were then incubated overnight at 4 °C with primary antibodies for cTnT (TNNT2 [1C11], Ab8295, dilution 1:250, Abcam, Cambridge, UK) and Ki67 (D3B5, dilution 1:400, Cell Signaling Technology, Danvers, MA, USA). Next, the cells were incubated with the corresponding secondary Alexa fluor-555 and −647 antibodies (1:400 dilution, Life Technologies, Carlsbad, CA, USA) for 1 h at room temperature. Nuclear DNA was labeled with DAPI. Images were acquired by an Eclipse Ti2 inverted microscope system (Nikon, Tokio, Japan). Z-stacks function was performed with 0.2 um thickness containing 11 frames under the 40×/0.95 NA objective in wide-field mode. All the images were processed afterwards for 3D deconvolution by NIS elements analysis software. Analysis and quantifications were performed with a maximum intensity projection by Image J.

### 2.5. Time-Lapse Imaging

Time-lapse sequence was implemented in order to keep track of hiPSC-CMs single cell cycle phases. hiPSC-CMs were replated on a Matrigel-coated 8-well chamber (Ibidi, Grafelfing, Germany) with a density of 1 × 10^5^ cells/well. The cells were treated with 3 µM CHIR99021 for 2 days, then subjected to time-lapse imaging on an Eclipse Ti2 inverted microscope system (Nikon, Tokio, Japan) for imaging under the objective of 10×/0.45 NA. Cells were kept at an Okolab enclosure with temperature 37 °C, 5% CO_2_ and 95% humidity. Each frame was acquired every 5 min until 12–24 h. Nuclear morphology varied in mitotic phases were captured from mononucleated or binucleated cells. To keep track of mCherry expression in hiPSC-CMs, The time-lapse sequence was developed 12 h post-transfection of hiPSC-CMs with the mCherry plasmid on an Eclipse Ti2 inverted microscope system (Nikon, Tokio, Japan) using bright-field mode/or confocal mode with a 10×/0.45 NA objective. Images were taken every 5 min for 12 h.

### 2.6. RNA Sequencing for CM Proliferation Genes

hiPSC-CMs collected at passage 1–3 and during maturation (day 77, 84 and 97) were obtained from a healthy donor hiPSC-CMs. RNA was isolated using ISOLATE II RNA Mini Kit (Meridian Biosciences, Cincinnati, OH, USA) according to the manufacturers’ instructions with minor adjustments. After the isolation of mRNA, libraries were prepared using the NEXTflexTM Rapid RNA-seq Kit (Perkin Elmer, Waltham, MA, USA). Libraries were sequenced on the Nextseq500 platform (Illumina, San Diego, CA, USA), producing single-end reads of 75 bp. Reads were aligned to the human reference genome GRCh37 using STAR v2.4.2a. Picard’s AddOrReplaceReadGroups v1.

(http://broadinstitute.github.io/picard/ (accessed on 4 June 2020)) was used to add read groups to the BAM files, which were sorted with Sambamba v0.4.5 and transcript abundances were quantified with HTSeq-count v0.6.1p1 using the union mode. Subsequently, reads per kilobase per million mapped reads (RPKMs) were calculated with edgeR’s RPKM function.

### 2.7. Transfection of hiPSC-CMs with mCherry

hiPSC-CMs were transfected (ViaFect (6:1), Promega, Madison, WI, USA) with 100 ng/cm^2^ PF256-H2B-mCherry and 20% FBS (Gibco). After 3 days, cells were harvested for FACS analysis, and fluorescent images were acquired with the EVOS (Life Technologies, Carlsbad, CA, USA). For quantification, nuclear expression (mCherry) was calculated as a percentage of positive nucleus to total nucleus (Hoechst 33342, Invitrogen, Waltham, MA, USA). The time-lapse imaging was developed with a 12 h post-transfection of hiPSC-CMs with mCherry plasmid on an LIPSI Ti2 inverted microscope system (Nikon, Tokio, Japan) using wide-field mode or confocal mode with a 10×/0.45 NA air objective. Images were taken every 5 min for 12 h.

### 2.8. FACS Analysis of Transfection Efficiency

After transfection, cells were dissociated via digestion with TrypLE™ Select (10×, Gibco, Waltham, MA, USA) and fixed in 4% paraformaldehyde (PFA). Next, immunostaining was performed on cells fixed with 4% paraformaldehyde (PFA) in PBS. Cells were incubated with anti-α-Actinin (clone A7811, Sigma Aldrich, Saint Louis, MO, USA) or anti-Ki67 (clone A833, Abcam, Cambridge, UK) labeled with Alexa-488 using 1:300 Alexa Fluor 488 (Thermo Fischer Scientific, Waltham, MA, USA) and 0.1 µg/mL Hoechst (33342, Invitrogen, Waltham, MA, USA). The staining was performed in PBS with 5% BSA (Roche) and 0.3% Triton-x-100 (Sigma Aldrich, Saint Louis, MO, USA). Stained cells were analyzed and sorted on an Astrios FACS (BD Pharmingen, San Diego, CA, USA). Data were collected from at least 10,000 events.

### 2.9. Statistics

Statistical analysis was conducted by Graphpad Prism software version 9. Data significance was statistically determined using an unpaired Student’s *t*-test for the comparison of two normally distributed datasets. One-way ANOVA or two-way ANOVA was performed with a Tukey’s multiple comparisons test to evaluate statistical differences among multiple datasets. Data plots are displayed as mean ± standard error of mean (sem), unless specified otherwise, and a *p*-value < 0.05 was set to determine statistical significance.

## 3. Results

### 3.1. Sarcomere Organization of hiPSC-CMs during Cell Cycle Phases

**Figure 1:**
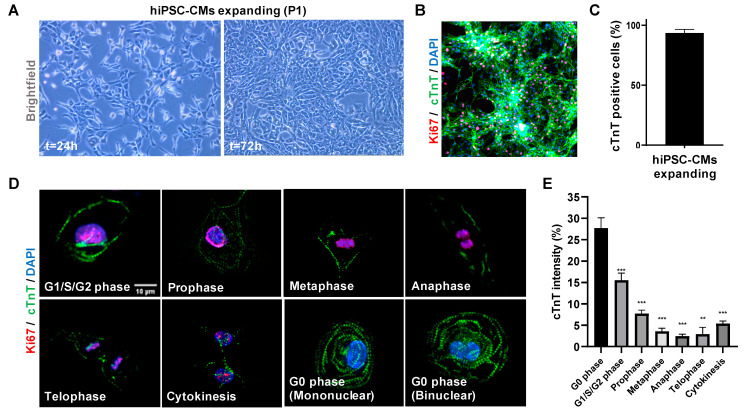
hiPSC-CMs sarcomere disassembly during mitosis. (**A**) Bright-field images of expanding hiPSC-CMs at passage 1 (P1). (**B**) Immunofluorescence for cardiac troponin T (cTnT), Ki67 and DAPI in hiPSC-CMs expanded with 3 µM CHIR99021 for 2 days. (**C**) Quantification of cTnT positive cells represented as a percentage of total cells (*n* = 3). (**D**) Representative immunofluorescence wide-field images of cTnT, Ki67 and DAPI during the different cell cycle phases of hiPSC-CMs. Note: during metaphase, anaphase and telophase the hiPSC-CM have a rolled-up morphology while contraction is absent. Scale bar indicates 10 µm. (**E**) Quantification of cTnT density analyzed by image J (*n* = 50). Bar plot represents mean cTnT intensity ± SEM. Significance assessed by unpaired *t*-test and defined by ** *p* < 0.01, and *** *p* < 0.001.

To visualize sarcomere organization during the cell cycle phases (G0, G1/S/G2 and M-phase), we differentiated hiPSC lines (Stanford Cardiovascular Institute 273 (SCVI-273 or SCVI-111) into CMs and performed imaging for sarcomere proteins and cell cycle marker expression (Ki67). Day 11 hiPSC-CMs were dissociated, replated and treated with glycogen synthase-3 beta (GSK3β) inhibitor to activate canonical Wnt signaling to maintain the proliferative capacity of early hiPSC-CMs [3,24] (Figure 1A). To confirm purity after passage 1 (P1), hiPSC-CMs were stained for cardiac troponin T (cTnT) and cell counting indicated the presence of >95% pure CMs (Figure 1B,C). High resolution confocal imaging of hiPSC-CMs, stained for Ki67 and cTnT, demonstrated that CMs in G0 phase and G1/S/G2 phase had the highest degree of organized sarcomeres and were spontaneously beating, while cells in M phase, especially during metaphase, anaphase and telophase, disassembled their sarcomeres, had a rolled-up morphology and were not lively contracting (Figure 1D,E and Figure S1). Interestingly, we observed that both mononuclear and binuclear hiPSC-CMs were found in G0 phase. Automated quantification of the amount of troponin T lines confirmed that quiescent CMs (G0) expressed more lines when compared to Ki67 positive CMs (*p* < 0.05). These results indicate that sarcomere breakdown, by reduced Troponin T lines, is present in hiPSC-CMs that undergo cytokinesis.

### 3.2. Live Tracing of Mitotic Figures in hiPSC-CMs

**Figure 2:**
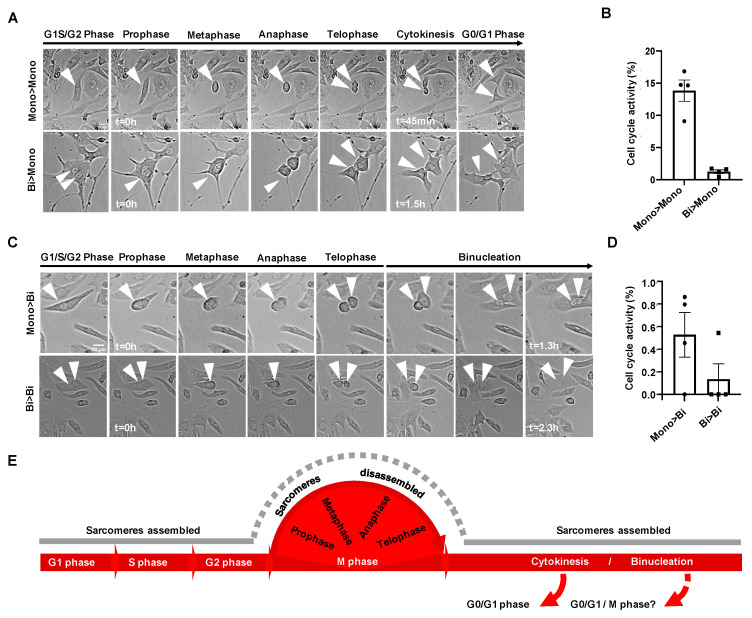
Time-lapse analysis of cytokinesis and binucleation in hiPSC-CMs. (**A**) Mononuclear (Mono > Mono) or binuclear (Bi > Mono) hiPSC-CMs undergoing cytokinesis after chromosomal segregation (rolled-up morphology). Time is indicated in hours (h). Scale bar indicates 20 µm. (**B**) Relative number of mitotic mononuclear (Mono > Mono) or binuclear (Bi > Mono) hiPSC-CMs undergoing cytokinesis. Average consists of 4 replicates including, in total, 105 mitotic cells of 771 counted cells for the SCVI-273 hiPSC line. Error barsindicate standard error (**C**) Bright-field time-lapse images showing binucleation and chromosomal segregation of preexistent mononuclear (Mono > Bi) or binuclear (Bi > Bi) hiPSC-CMs. Time is indicated in hours (h). Scale bar indicates 20 µm. (**D**) Relative number of mitotic mononuclear (Mono > Bi) or binuclear (Bi > Bi) hiPSC-CMs undergoing binucleation. Average consists of 4 replicates including, in total, 105 mitotic cells of 771 counted cells for the SCVI-273 hiPSC line. Error bars indicate standard error. (**E**) Schematic diagram visualizing cell cycle in relation to status of the sarcomeres.

To assess longitudinal characterization of CMs in M-phase (segregation of chromosomes) and their progeny, we recorded time-lapse videos of proliferating SCVI-273 hiPSC-CMs and analyzed the fraction of cells that went through the M-phase, indicated by a rolled-up morphology during the anaphase [13]. We identified that both mononuclear (~13% of total) and binuclear cells (~1.5% of total) can enter the M-phase to segregate their chromosomes to give rise to two daughter cells (Figure 2A,B) (Videos S1 and S2). Interestingly, binuclear CMs also (re)entered the M phase instead of directly providing one nucleus to each daughter cell (Figure 2A, arrows in lower panel).

Moreover, we observed that both mononuclear (~0.5% of total) and binuclear (~0.1% of total) hiPSC-CMs entered the M-phase to become or remain binuclear (Figure 2C,D) (Videos S3 and S4). We repeated these experiments in SCVI-111 hiPSC-CMs treated with CHIR99021 and found that all four sequences were present with similar distributions, the binuclear-to-binuclear transition being the rarest event (Figure S2). Given our study (Figure 1) on sarcomere density during M-phase it appears that for each form of nuclear or cytoplasmic division of hiPSC-CMs, cells underwent chromosomal segregation accompanied by sarcomere breakdown (Figure 2E).

### 3.3. Gene Expression Analysis of Long-Term Proliferating, Matured Non-Proliferative hiPSC-CMs and Adult Human Heart Tissue

**Figure 3:**
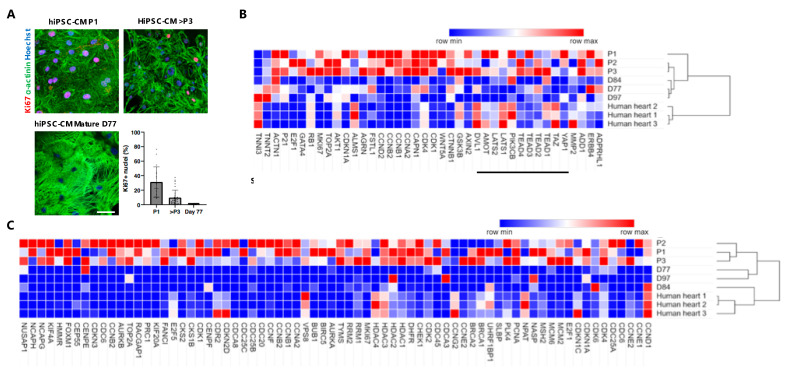
Selected gene expression profiles for developmental growth and cell cycle regulators in serially passaged immature hiPSC-CMs and metabolic matured hiPSC-CMs. (**A**) Representative immunofluorescence for Hoechst (blue), Ki67 (red), and a-actinin (green) in hiPSC-CMs treated with CHIR99021 at passage 1 (P1) and 4 (*p* > 3), versus metabolic matured hiPSC-CMs at day 77. Graph displays the percentage of Ki67 positive nuclei per condition (P1, >P3 or day 77) (*n* = 3 differentiations with 17, 32 and 24 quantified images). (**B**) Heatmap showing the expression patterns of 40 selected genes involved in the proliferation and/or development of hiPSC-CMs between expanding hiPSC-CMs (passage 1–3), metabolic matured hiPSC-CMs (day 77, 84 and 97) and adult human heart tissue. (**C**) Heatmap showing the expression patterns of cell cycle phase-related genes between expanding hiPSC-CMs (passages 1–3), metabolic matured hiPSC-CMs (day 77, 84 and 97) and adult human heart tissue. Red indicates high expression and blue indicates low expression of represented genes. Heatmaps are clustered and show the RPKMs for the selected genes.

To provide more background on the cell cycle status of immature hiPSC-CMs, which were long-term cultured with GSK3β inhibitor CHIR99021 and sequential serially passaged (up to P4), we performed gene expression profiling for each serial passage (P1 (day ~18), P2 (day ~24) and P3 (day ~35)) and compared this to long-term metabolic matured hiPSC-CMs of day 77, 84 and 97. Confirmative immunohistochemistry for α-actinin and Ki67 demonstrated that proliferation rates (Ki67 positivity) were decreased when reaching a higher passaging number in expanding CMs [25] (Figure 3A). In contrast, in long-term metabolic matured hiPSC-CMs, we observed almost complete absence of proliferative cells [25] (Figure 3A). Moreover, higher organization of sarcomeres was visible in mature versus immature hiPSC-CMs (Figure 3A, lower left panel).

Whole genome RNA sequencing revealed that the expression of sarcomere genes in mature CMs and human heart tissue, such as TNNI3 and TNNT2, were inversely correlated to genes associated with proliferation, such as TOP2A, MKI67 and CCND2 (Figure 3B). The opposite phenomenon was observed in the gene expression of proliferative CMs from P1, P2 and P3. Furthermore, genes associated with embryonic signaling pathways, including Wnt and Hippo, were upregulated in proliferating hiPSC-CMs when compared to quiescent CMs (Figure 3B). Analysis of M-phase, G2 phase, S phase and G1 phase-related genes showed an increase in the long-term Wnt-treated hiPSC-CMs from P1 to P3 versus the metabolic maturated CMs from day 77, 84 and 97 and adult human hearts (Figure 3C). Altogether, these data showed that in hiPSC-CMs, the cell cycle gene expression is inversely correlated to sarcomere gene expression and maturity of CMs.

### 3.4. GSK3β Inhibition Increases Non-Viral Vector Incorporation Efficiency in hiPSC-CMs

**Figure 4:**
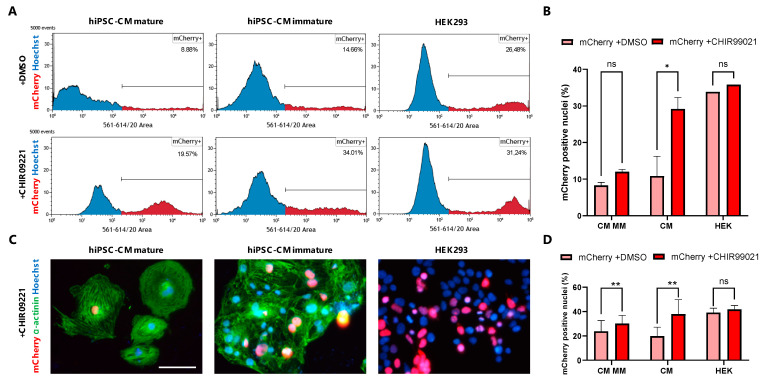
Transfection efficiency in immature hiPSC-CMs vs. mature hiPSC-CMs and HEK293 cells. (**A**) Quantitative flow-cytometry graph indicating high transfection efficiency of up to 34% in immature and up to 20% in mature hiPSC-CM treated with 2 µM of CHIR99021, 100 ng/mL mCherry plasmid and with ViaFect transfection reagent. (**B**) Bar graph displaying mean mCherry transfection efficiency in immature (CM) and mature (MM) hiPSC-CMs and HEK293 cells (HEK) treated with CHIR99021 or DMSO carrier control in the presence of ViaFect or Lipofectamin transfection reagent. Mean flow-cytometry values of 3 biological replicates are represented and error bars indicate standard deviation. (**C**) Representative immunofluorescence for Hoechst (blue), mCherry (red) and α-actinin (green) after 72 h of transfection treatmen (ViaFect)t in hiPSC-CMs and HEK293 cells treated with CHIR99021. (**D**) Quantitative bar graph representing mean transfection efficiency in a fraction of hiPSC-CMs or HEK293 cells by mCherry expression of high transfection efficiency. Experiments performed 3 biological replicates. Error bars indicated standard deviation. * indicates *p* < 0.05, ** indicates *p* < 0.01).

The efficiency of non-viral vector incorporation in low-proliferative hiPSC-CMs is usually below 20%, which highly challenges its usefulness in, e.g., molecular knockdown experiments [26]. We reasoned that GSK3β inhibition in hiPSC-CMs, promoting a high-proliferative status, would also potentially enhance the incorporation and expression of introduced plasmid DNA. To this end, we used day 30 high-proliferative hiPSC-CMs treated with CHIR99021 and low-proliferative non-GSK3β inhibition treated age-matched controls and transfected these for 72 h with a PF256-H2B-mCherry plasmid (mCherry expression controlled by the histone cluster 1 promotor in the presence of Lipofectamine or ViaFect transfection reagent [27]. Non-viral vector incorporation efficiency was measured by flowcytometry for mCherry and Hoechst and fluorescence-based immunohistochemistry for α-actinin and expression of mCherry 72 h after the start of transfection (Figure S3A–C). We observed that in the presence of CHIR99021 30.5 ± 4.9% of day 30, high-proliferative hiPSC-CMs expressed mCherry versus 10.8 ± 5.4% (*p* = 0.03) of low-proliferative hiPSC-CMs cultured without a GSK3β inhibitor (Figure 4A). To investigate the cell cycle-independent effects of the GSK3β inhibition of non-viral vector incorporation efficacy, we also transfected day ~80 metabolic matured and non-proliferative hiPSC-CMs in the presence or absence of CHIR99021. We found that in the presence of CHIR99021, non-proliferative CMs expressed mCherry in 12 ± 0.6%, while in the absence of CHIR99021, matured CMs were mCherry positive in 8.3 ± 0.8% (*p* = 0.89) (Figure 4B). Additionally, we compared ViaFect with Lipofectamine 3000 transfection reagent and found that CHIR99021 treatment increased the transfection efficiency in both reagents by more than 50% (Figure S3D). To confirm general transfection efficiency and potential non-cell cycle-dependent effects of CHIR99021, we utilized a common source of indefinitely proliferating HEK293 cells and transfected these with and without GSK3β inhibition. In HEK293 cells we observed no significant increase in transfection efficiency when CHIR99021 was added to the media versus the DMSO carrier control (33.8% vs. 35.8%, *p* = 0.93) (Figure 4C). Next, we confirmed the expression of mCherry in the fraction of CMs, which were treated with CHIR99021 via immunohistochemistry for α-actinin and endogenous mCherry fluorescence (Figure 4D). These data illustrate that CHIR99021 promotes non-viral vector uptake in hiPSC-CMs predominantly via a cell cycle-dependent way.

### 3.5. Live Tracing of Non-Viral Vector-Based Fluorescence Expression in hiPSC-CMs

**Figure 5:**
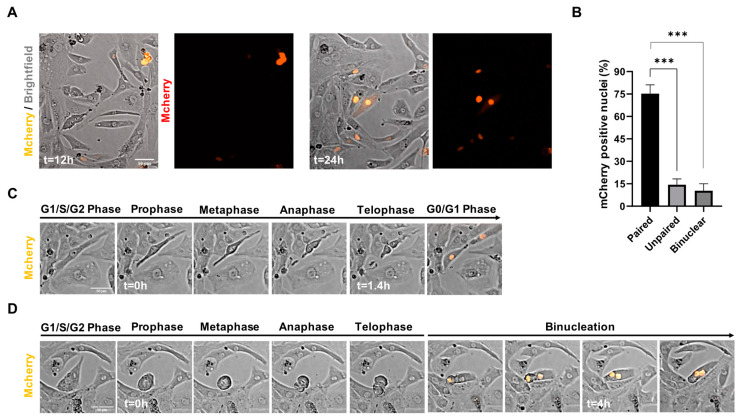
Time-lapse analysis of non-viral vector mCherry incorporation in proliferating hiPSC-CMs. (**A**) Representative time-lapse images of the mCherry positive hiPSC-CMs 12 h and 24 h post-transfection with mCherry-H2B plasmid and ViaFect transfection reagent. (**B**) Quantification of the mCherry positive paired, unpaired and binucleated cells represented in percentages (*n* = 100). Data are shown as mean ± SEM. *** *p* < 0.001 as calculated by Student’s *t*-test. (**C**) Selected time-lapse images visualizing mCherry expression in a mononuclear hiPSC-CM undergoing cytokinesis. (**D**) Selected time-lapse images demonstrating mCherry expression after binucleation of a mononuclear hiPSC-CM. Time is indicated in hours (h). Scale bar indicates 50 µm.

Based on our data, it appears that the cell cycle-related effects of CHIR99021 are the most crucial for the transfection efficiency in hiPSC-CMs (Figure 4). To demonstrate that indeed the active cycling hiPSC-CMs were to express the mCherry plasmid first, we recorded time-lapse videos in bright-field and immunofluorescence mode for 12 h starting at 1-day post-transfection of the cells. After 24 h and 36 h of transfection we observed a pattern of mainly neighboring cells that started to express the mCherry fluorescent protein (Figure 5A). Quantification of neighboring cells confirmed that 75.25% were paired mCherry positive cells, while 14.35% of mCherry positive cells were unpaired and 10.4% were binucleated cells (Figure 5B). Next, we analyzed our time-lapse videos for cells that showed a rolled-up morphology during mitosis and found that the fraction of cells undergoing cytokinesis started to express mCherry protein mainly right after cytokinesis had occurred (Figure 5C). In cells that became binucleate after mitosis we noticed an expression of mCherry in both nuclei upon completion of the binucleation process (Figure 5D). These data indicate that incorporation of vectors occurs before or during M-phase, while expression of the PF256-H2B-mCherry vector starts immediately after the M-phase.

## 4. Discussion

The cardiomyocyte compartment of the human heart is largely non-mitotic and with an annual cell turnover lower than 1%, this explains the lack of true regenerative capacity [28]. In the neonatal period, CMs still proliferate and the heart possesses a capacity to regenerate upon injury, while in the adult myocardium the rate of CM self-renewal becomes too low to compensate for potential cell loss by, e.g., myocardial infarction. In mammals, cardiac growth by hyperplasia changes to hypertrophy early after birth, coinciding with increasing DNA content of the CMs via multinucleation and nuclear polyploidization [11]. Previous studies have shown a correlation between the amount of diploid CMs and substantial regenerative capacity, raising the hypothesis that multinucleation and/or polyploidy forms a block for CM proliferation and heart regeneration [9,10,25,29,30]. On the contrary, there is evidence for self-duplication of multinucleated CMs, suggesting a more complex situation of ploidy in relation to cardiac repair [31,32]. For this study, we utilized time-lapse recordings to study the sequence of sarcomere distribution during mitosis, followed by cytokinesis, multinucleation or self-duplication in massively expanding hiPSC-CMs.

In Figure 1 we demonstrate that during mitosis of hiPSC-CMs, sarcomere breakdown is predominantly activated during the metaphase, anaphase and telophase and cells transiently stop contracting during cytokinesis. After cytokinesis and the following G0 or G1/S/G2 phases, the sarcomeres are restored and spontaneous beating is reinitiated. Moreover, we observe that in hiPSC-CMs the sequence of mitosis is followed by cytokinesis and self-duplication versus multinucleation (Figure 2). Remarkably, for all of these sequences, we noticed that chromosomal segregation (M-phase), accompanied by sarcomere disassembly were both present during mitosis (Figure 1 and Figure 2). Mechanistically, we show that Wnt and Hippo genes are highly expressed in the high-proliferative juvenile hiPSC-CMs versus non-proliferative metabolic matured CMs (Figure 3). In addition, we demonstrate in Figure 4 that high-proliferative hiPSC-CMs in G1/S/G2 or M phase can incorporate non-viral vectors at a much higher degree than low-proliferative or non-proliferative hiPSC-CMs. Our data also indicates that the earliest expression of such vectors is present in CMs that just have gone through M phase/chromosomal segregation (Figure 5).

Collectively, our study provides information on sarcomere density during mitosis and sequential cytokinesis, self-duplication or multinucleation. Our live-tracing studies (Figure 2 and Figure S2) indicated that cell cycle activity of hiPSC-CMs was varying among different cell lines. We observed that cytokinesis occurred in 13–40%, self-duplication in 1.5–2.5% and multinucleation in 0.6–1.7% of events. Multinucleation as a result of a binuclear cells going through the M phase formed a rare event (0.1–0.2%). The observation that binucleated cells can self-duplicate is in line with previous studies in mice that have found that regenerative capacity also originates from preexisting and multinucleated CMs [31,32]. The occurrence of binucleation, however, is 5–10 times lower than self-renewal in our hiPSC-CM system, in which we concurrently activate Wnt signaling and remove cell–cell contacts (Figure 2B,D and Figure 5B). Therefore, this higher incidence of cytokinesis from mononucleated cells in our system also appears to be in line with the concept that diploid cells are the most powerful for endogenous heart regeneration [10]. The observation that hiPSC-CMs disassemble their sarcomeres followed by temporary quiescence is potentially explanatory for the low number of cell division in the adult human heart, which cannot allow itself “to skip a beat”.

Besides the heart, the process of multinucleation as a result of acytokinetic cell division also occurs in skeletal muscle and forms of cancer. Previous studies have demonstrated that this multinucleation process is reflected in hiPSC-derived muscle progenitor and cancer cell line models [33,34,35].

Additionally, our non-viral vector gene transfection studies show that efficiency is highly related to the proliferative status of hiPSC-CMs. Although multiple methods of genetic modifications exist (i.e., lipofectamine-mediated transfection, and viral-based transduction), their efficiency, cytotoxicity, safety, and cost remain unsatisfactory and often below 10–20% [26]. We used our live hiPSC-CM imaging setup to study the transfection in proliferating (canonical Wnt activation) versus non-proliferating hiPSC-CMs. We found that hiPSC-CMs after chromosomal segregation (mitotic figure) were the first cells expressing a non-viral vector-based fluorescent protein. A smaller fraction of cells incorporates non-viral vector DNA in a cell cycle-independent manner. This improved approach for gene transfection is directly usable for research involving molecular studies and cardiac tissue engineering applications.

Several limitations should be considered when interpreting the results of our study. Although we used expanding hiPSC-CMs reaching purity of up to 98%, for our time-lapse videos we were not able to live trace sarcomere, and therefore, there is potentially a risk of other cell types being recorded and analyzed. Secondly, CHIR99021, a potent GSK3β inhibitor present in our expansion culture system, could potentially also have non-cell cycle-related effects leading to increased transfection rates. Our data in Figure 5, however, indicates that activation of the cell cycle and M phase is needed for early incorporation of non-viral vectors. Lastly, the lack of environmental cues in our in vitro system put limitations on the interpretation of the described event of self-duplication as a potential route for heart regeneration.

This study demonstrates that there is a complex landscape in hiPSC-CM proliferation, multinucleation and self-duplication. Moreover, enhanced incorporation of non-viral vectors is related to Wnt activation and cell cycle activity of hiPSC-CMs, which forms a strong tool for molecular gene studies in hiPSC-CMs and cardiac tissue engineering.

## Supplementary Materials

The following are available online at https://www.mdpi.com/article/10.3390/jcdd9020043/s1, Figure S1: Immunofluorescence channels in mitotic hiPSC-CMs. The 594-channel (red) represents Ki67, the 488 nm channel (green) represents cardiac troponin T (cTnT) and the 350 nm channel (blue) represents all nuclei (DAPI), Figure S2: Time lapse analysis of cytokinesis and binucleation in hiPSC-CMs. (**A**) Mononuclear (Mono > Mono) or binuclear (Bi > Mono) hiPSC-CMs undergoing the cytokinesis after chromosomal segregation (rolled-up morphology). Time is indicated in hours (h). Scale bar indicates 20 um. (**B**) Average relative number of mitotic mononuclear (Mono > Mono) or binuclear (Bi > Mono) hiPSC-CMs undergoing cytokinesis. Average consists of 4 replicates including in total 564 mitotic cells of 1456 counted cells for the SCVI-111 hiPSC line. Error bars indicate standard deviation (**C**) Bright field time-lapse images showing binucleation and chromosomal segregation of preexistent mononuclear (Mono > Bi) or binuclear (Bi > Bi) hiPSC-CMs. Time is indicated in hours (**h**). Scale bar indicates 10 um. (**D**) Relative number of mitotic mononuclear (Mono > Bi) or binuclear (Bi > Bi) hiPSC-CMs undergoing binucleation. Average consists of 4 replicates including in total 564 mitotic cells of 1456 counted cells for the SCVI-111 hiPSC line. Error bars indicate standard error, Figure S3: Flowcytometry strategy and transfection efficiency between different reagents. (**A**,**B**) Gating strategy to select nuclei posi-tive for Hoechst and/or mCherry and/or alpha-actinin-488 conjugated fluorescence. (**C**) Non-transfected hiPSC-CMs and HEK293 cells for negative control. (**D**) Side-to-side comparison of ViaFect and Lipofectamin 3000 (Lipo3k) transfection reagents in hiPSC-CMs transfected with mCherry plasmid in the presence or absence of 2 uM CHIR99021, Video S1: Mononuclear cell division of hiPSC-CMs (Mono > Mono). Video S2: Binuclear self-duplication of hiPSC-CMs (Bi > Mono). Video S3: Binucleation from mononucleated hiPSC-CMs (Mono > Bi). Video S4: Binucleation from binucleated hiPSC-CMs (Bi > Bi).
