# Supplementary material for: Sarcomere Disassembly and Transfection Efficiency in Proliferating Human iPSC-Derived Cardiomyocytes
Source: J Cardiovasc Dev Dis. 2022 Jan 27;9(2):43. doi: 10.3390/jcdd9020043 (PMC8880351; doi:10.3390/jcdd9020043)
Supplement: Supplementary file 1 [file jcdd-09-00043-s001.zip › jcdd-1425009-supplementary.pdf]

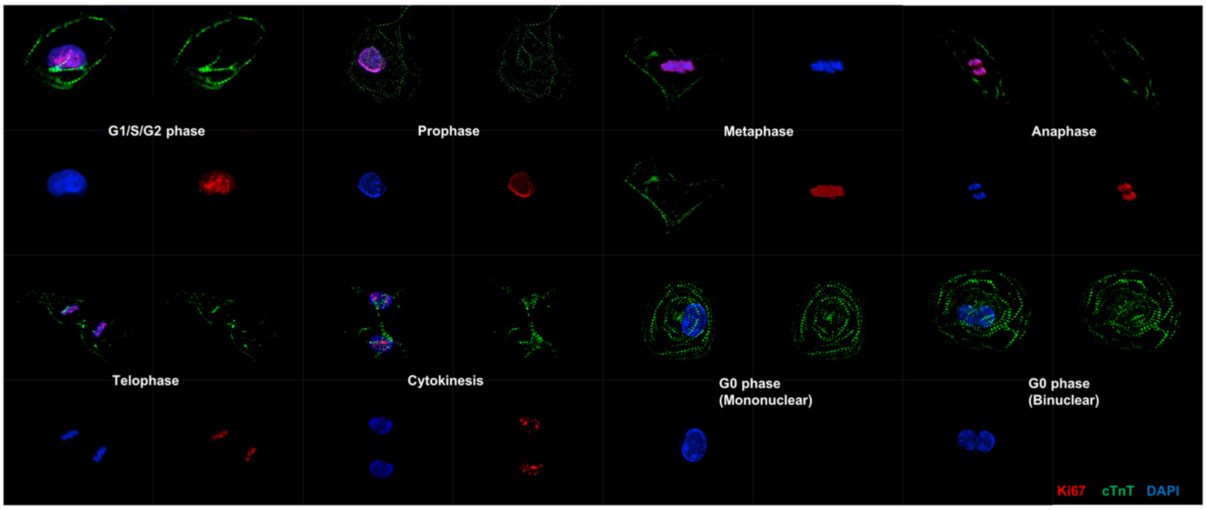

Figure S1: Immunofluorescence channels in mitotic hiPSC-CMs. The 594-channel (red) represents Ki67, the 488nm channel (green) represents cardiac troponin T (cTnT) and the 350nm channel (blue) represents all nuclei (DAPI).

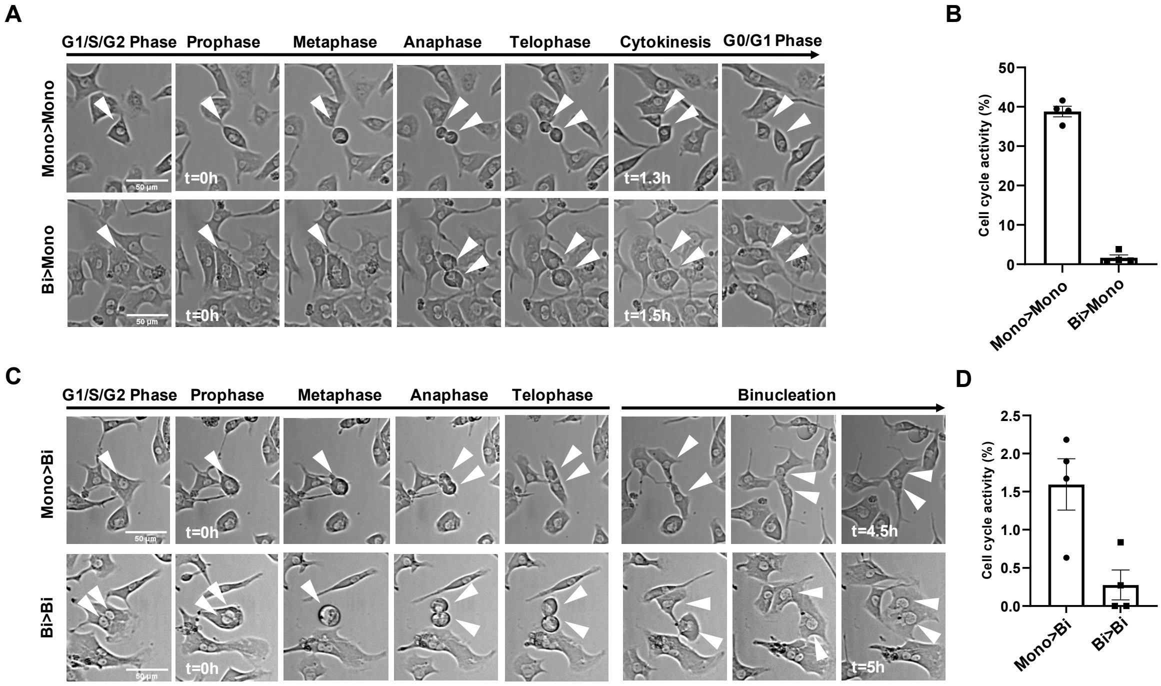

Figure S2: Time lapse analysis of cytokinesis and binucleation in hiPSC-CMs. (**A**) Mononuclear (Mono>Mono) or binuclear (Bi>Mono) hiPSC-CMs undergoing the cytokinesis after chromosomal segregation (rolled-up morphology). Time is indicated in hours (h). Scale bar indicates 20 um. (**B**) Average relative number of mitotic mononuclear (Mono>Mono) or binuclear (Bi>Mono) hiPSC-CMs undergoing cytokinesis. Average consists of 4 replicates including in total 564 mitotic cells of 1456 counted cells for the SCVI-111 hiPSC line. Error bars indicate standard deviation (**C**) Bright field time-lapse images showing binucleation and chromosomal segregation of preexistent mononuclear (Mono>Bi) or binuclear (Bi>Bi) hiPSC-CMs. Time is indicated in hours (h). Scale bar indicates 10 um. (**D**) Relative number of mitotic mononuclear (Mono>Bi) or binuclear (Bi>Bi) hiPSC-CMs undergoing binucleation. Average consists of 4 replicates including in total 564 mitotic cells of 1456 counted cells for the SCVI-111 hiPSC line. Error bars indicate standard deviation.

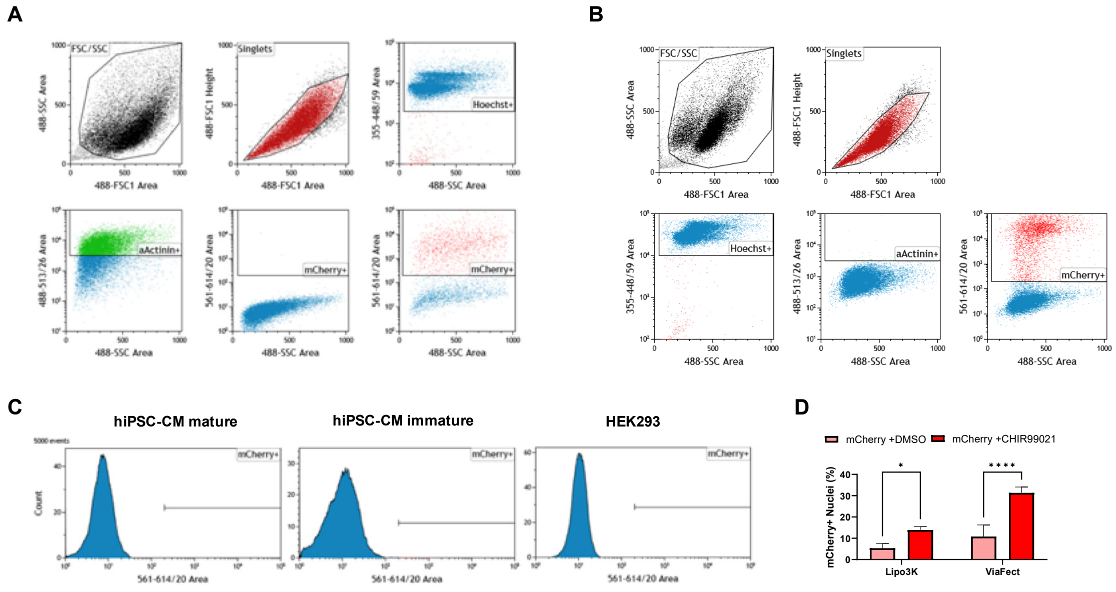

Figure S3: Flowcytometry strategy and transfection efficiency between different reagents. **(A-B)** Gating strategy to select nuclei positive for Hoechst and/or mCherry and/or alpha-actinin-488 conjugated fluorescence. (**C**) Non-transfected hiPSC-CMs and HEK293 cells for negative control. (**D**) Side-to-side comparison of ViaFect and Lipofectamin 3000 (Lipo3k) transfection reagents in hiPSC-CMs transfected with mCherry plasmid in the presence or absence of 2uM CHIR99021.

Video S1: Mononuclear cell division of hiPSC-CMs (Mono>Mono).

Video S2: Binuclear self-duplication of hiPSC-CMs (Bi>Mono).

Video S3: Binucleation from mononucleated hiPSC-CMs (Mono>Bi).

Video S4: Binucleation from binucleated hiPSC-CMs (B>Bi).
